# Safeguarding the right to collective bargaining in the face of precarious self-employment – Gaps in Protection in Austria and Germany and obligations under European law

**DOI:** 10.1177/20319525251390885

**Published:** 2025-11-10

**Authors:** Franziska Pupeter, Karin Lukas, Carolina Bertazolli

**Affiliations:** PhD candidate at the Legal Studies Department, at 47797 Central European University, Wien, Austria; Senior Visiting Researcher and Professor at the Legal Studies Department, at CEU, Wien, Austria; PhD candidate at the Legal Studies Department, at CEU, Wien, Austria

**Keywords:** Solo self-employment, platform work, human rights, freedom of association, right to collective bargaining, trade unions, comparative law

## Abstract

In recent years, terms like platform work, gig work and cloud work have sparked a debate amongst labour lawyers and lawmakers. Even though the circumvention of traditional employment relationships is not a new phenomenon, technological change and the increasing flexibility of work put previously established standards on fair wages and just working conditions anew at stake. Trade Unions have traditionally played a key role in protecting those whose livelihoods depend on providing labour for others. A closer look at the existing legal frameworks in Austria and Germany shows that solo self-employed persons, meaning workers who do not have an employment contract and do not employ others, can benefit only to a very limited extent from the capacity of trade unions to bargain collectively, which is a critical instrument to ensure fair working conditions, including fair wages. This article argues that the European human rights standards require national legislators to take active steps in ensuring the right to bargain collectively for every person in need. In section I, we will start by introducing the central concepts of our analysis and discuss the extent to which the legal frameworks in Austria and Germany allow for collective bargaining on behalf of solo self-employed persons. In section II, we will look at several layers of human rights protection at the European level, with a focus on the personal scope of the right to collective representation. In the final section, we will address the interplay between human rights standards and EU law, before concluding with our recommendations for domestic legal change in Austria and Germany.

## I. Introduction

In recent years, terms like platform work, gig work and cloud work have sparked a debate amongst labour lawyers and lawmakers. Even though the circumvention of traditional employment relationships is not a new phenomenon, technological change and the increasing flexibility of work put previously established standards on fair wages and just working conditions anew at stake. Trade Unions have traditionally played a key role in protecting those whose livelihoods depend on providing labour for others. A closer look at the existing legal frameworks in Austria and Germany shows that solo self-employed persons, meaning workers who do not have an employment contract and do not employ others, can benefit only to a very limited extent from the capacity of trade unions to bargain collectively, which is a critical instrument to ensure fair working conditions, including fair wages. This article argues that European human rights standards require national legislators to take active steps in ensuring the right to bargain collectively for every person in need.

In section I, we will start by introducing the central concepts of our analysis and discuss the extent to which the legal frameworks in Austria and Germany allow for collective bargaining on behalf of solo self-employed persons. In section II, we will look at several layers of human rights protection at the European level, with a focus on the personal scope of the right to collective representation. In the final section, we will address the interplay between human rights standards and EU law, before concluding with our recommendations for domestic legal change in Austria and Germany.

## II. Collective bargaining by solo self-employed persons in Austria and Germany

In the Austrian and the German legal systems, an employment relationship is the precondition for being classified as an employee (in German: ‘*Arbeitnehmer:in*’) and benefitting from worker protection laws.^1^ However, the written contract, on which a work relationship is based, is not the sole indicator of the existence an employment relationship. Even if it is not formulated as an employment contract, it may still be classified as such if the factual situation meets the criteria for an employment relationship.^2^ Therefore, one may speak of ‘false self-employed persons’ for workers whose contracts indicate that they are self-employed whilst, according to a correct legal classification, they would be employed. In this article, however, we assume that genuine self-employed workers may also be in need of protection. Thus, we will refer to workers in a broader sense, which also includes genuinely self-employed persons.

Given the heterogeneity of the group of self-employed persons, we note that not all of them are necessarily in precarious working situations. For example, doctors, lawyers and IT experts belong to professional groups that secure a high income in their capacity as self-employed workers. However, others are in a weak negotiating position *vis-á-vis* their contractual partners from the outset, and thus enter into contractual relations with little remuneration. These workers may be considered economically dependent in the sense that they work for one or very few contractors and have a low income. In the following, we will mainly refer to ‘solo self-employed persons’ which is the central category for this article. Thereby, we mean workers who do not have a (formal) employment contract and do not employ others. Since solo self-employed persons rely primarily on their own personal labour to maintain their income, we assume that they are likely to be in a weak negotiating position.^3^

### A. Number of solo self-employed persons in Austria and Germany

The public agency *Statistik Austria* estimated that there were 502,600 self-employed persons in Austria in 2024, which accounts for 11.2% of the working population.^4^ The group of solo self-employed persons, defined as not having any employees, comprised 307,800 workers.^5^ Thus, more than half of the self-employed persons in Austria were solo self-employed. In an older study from the year 2017, the agency outlined a group of persons with three cumulative characteristics of not having any employees, working for only one contractor and having this contractor determine their working hours.^6^ This narrowly defined group comprised 11,400 persons in Austria. Unfortunately, these studies did not provide any information on the income of these groups.

In Germany, the Federal Ministry for Labour and Social Affairs commissioned a study to provide figures for the year 2022. As a country with a working population roughly 10 times the size of that of Austria, it counted a total of 3.6 million self-employed persons. This included 1.8 million solo self-employed persons.^7^ The study also shows that, in the year 2021, the median of the monthly net income of solo self-employed persons in Germany was 1,700 euros, and therefore considerably lower than the median income of the self-employed person in general (3,000 euros).^8^ From these figures, we derive that solo self-employed persons are indeed more likely to be economically dependent than self-employed persons who have employees. Furthermore, we derive a need to guarantee a decent income for solo self-employed workers by means of collective bargaining. In the next section, we analyse the extent to which the legal frameworks in both countries allow solo self-employed workers to benefit from collective bargaining by the trade unions.

### B. A comparison of legal frameworks on the social partnership

Despite the many parallels in Austrian and German labour laws, there are some substantial differences with regard to guaranteeing collective representation. According to the Austrian Confederation of Trade Unions (hereinafter: ÖGB), 94% of employed persons in Austria are covered by a collective agreement.^9^ In comparison, only 49% of German employees benefitted from a collective agreement in 2023.^10^

These vastly different figures correspond to the following divergencies in the legal realm: Firstly, Germany, unlike Austria, has a general statutory minimum wage.^11^ In Germany, wages fixed in collective agreements are complementary to the minimum benchmark set by statutory law, whereas in Austria, employees rely only on the standards set by collective bargaining. Secondly, the legal frameworks in Austria and in Germany differ with regard to the personal scope that they prescribe for collective agreements. According to the German Collective Bargaining Act (hereinafter: TVG), collective agreements bind only the collective bargaining parties,^12^ meaning trade unions and employers’ associations. They have normative power only for the members of the trade unions and employers’ associations. Since membership is not mandatory, they do not apply to every worker and employer.^13^

In contrast, the Austrian system of social partnership obliges businesses and employers to be members of the Chamber of Commerce^14^ and the trade unions enter collective agreements with the sector-specific units of the Chamber.^15^ The mandatory membership effectuates great leverage in combination with the ‘outsider effect’ prescribed by law. The Austrian Labour Constitution Act states that all employees can rely on a collective agreement even if they are not themselves members of the relevant trade union. It suffices that the employer is a member of the Chamber of Commerce.^16^

To summarise, there are systemic differences in the legal framework, which explains why a much higher percentage of Austrian employees are covered by collective agreements. Nevertheless, collective bargaining is equally crucial for solo-entrepreneurs in both countries since the statutory minimum wage in Germany applies only to employed persons.^17^ In that sense, it is important to keep in mind that the overall density of collective agreements in Germany is lower than that in Austria.

### C. A comparison of legal categories for solo self-employed persons

Despite these idiosyncrasies in the set-up of the social partnership, Austrian and German labour law are very similar in other aspects. They are both built, in principle, on the dichotomy between employment and self-employment. In partial deviation from this dichotomy, both legal systems foresee two in-between categories that are often discussed in light of the protection of self-employed persons: the category of ‘homeworkers’ and that of ‘employee-like persons.’ These concepts exist as legal categories in both countries and also apply to solo self-employed persons. However, they differ in their scope.

The Austrian law defines ‘homeworkers’ as those who perform tasks from home that are ordered by a contractor, including the manufacturing, processing or packing of goods.^18^ This category includes self-employed persons, but its scope of application is rather limited because these tasks are no longer performed from home, at least not in industrialised countries like Austria. In contrast, the German law on homeworkers does not specify the tasks performed.^19^ Its scope of application is therefore broader and may also include self-employed persons in the digital sector.^20^

Regarding the legal category of ‘employee-like persons’, the differences between the two countries are less striking. The German Collective Bargaining Act defines employee-like persons as those who are economically dependent from their contractor(s) and comparable to employees in their need for social protection.^21^ In Austria, the notion has been shaped by judicial interpretation, but is nevertheless similar to that in Germany: employee-like persons are economically-dependent self-employed persons, typically characterised as relying on their own personal labour for income, as not having employees themselves, and earning only a small income.^22^ In light of these definitions, we consider that the legal category of ‘employee-like persons’ largely correlates with the group of ‘economically dependent solo self-employed workers’ as recorded by the statistics referenced above.

### D. Collective bargaining by solo self-employed persons

Having clarified these categories, we are now closing in on the central question: Does national law in Austria and Germany allow solo self-employed persons to benefit from the minimum wages fixed in collective agreements? Both legal systems are somewhat inconclusive on this question.

The German TVG stipulates, in section 1, that collective agreements may establish rules regarding employment relationships, which excludes contractual relationships that self-employed persons enter into with their business partners. However, section 12a TVG expressly includes the category of employee-like persons. Therefore, in principle, the German law allows for collective bargaining on behalf of economically dependent solo self-employed persons. Nevertheless, the literature notes that not all solo self-employed persons may benefit due to the strict interpretation of the criteria according to which a self-employed person is considered ‘economically dependent’: It is required that they work primarily for one contractor or receive at least 50% of their total income from one contractor.^23^ The second exception in German law concerns the category of homeworkers. Whilst they are generally excluded from the TVG, the German Law on Homeworkers foresees the possibility of written agreements between homeworkers’ associations and their clients.^24^ These written agreements are to be regarded as akin to collective agreements ‘by virtue of fiction.’^25^ Again, the question remains whether the category of homeworkers comprises all solo self-employed persons. The judicial interpretation is ambiguous on the questions of whether and in what sense the legal definition of homeworkers requires them to be economically dependent in order to benefit from collective bargaining.^26^

In contrast, the Austrian Labour Constitution Act does not provide for collective bargaining on behalf of employee-like persons.^27^ Even though the Austrian Law on Homeworkers also foresees the possibility for homeworkers to bargain collectively,^28^ it does not offer a real option for those solo self-employed homeworkers who do not work in the production, processing and packaging of goods.^29^

The Austrian legal system foresees the possibility for self-employed collective bargaining in cases where a separate sector-specific law has been created. For example, for freelance journalists and other self-employed workers in the media sector, the Journalist Work Act explicitly foresees this possibility^30^ and is the legal basis for a collective agreement that benefits self-employed writers and journalists in Austria.^31^ Self-employed journalists in Germany, on the other hand, benefit from a collective agreement that is based on section 12a TVG on employee-like persons.^32^ Whereas the Austrian law effectively blocks the creation of collective agreements for the solo self-employed, apart from the exceptions that apply to homeworkers and journalists (and from 2026, to persons with freelance contracts), German law allows for it. Nevertheless, there are still only a few collective agreements for the solo self-employed in Germany,^33^ which may be due to the overall weaker system of social partnership and the low density of collective agreements in general. We can conclude that in both countries, the law allows for collective bargaining agreements that benefit solo self-employed persons only to a very limited extent.

## III. Collective bargaining as a human right at a European level

Having compared the collective bargaining systems in Austria and Germany as well as the degree to which national labour law allows solo self-employed persons to benefit from collective bargaining, we set out now to discuss the legal standards of collective bargaining as a human right. We will take a closer look at the rights provisions that guarantee the freedom of assembly and the freedom to join and form trade unions, as specified by some human rights documents. These rights have an individual dimension - the individual's right to join an assembly or trade union - as well as a collective dimension that protect trade unions as such. The right to form and join trade unions is a necessary precondition for the right to bargain collectively, which will be at the centre of our analysis. We will examine the two main human rights documents within the framework of the Council of Europe, namely, the European Convention of Human Rights and the European Social Charter, as well as the Charter of Fundamental Rights of the European Union. With regard to each of these documents, we will clarify whether they prescribe the right to bargain collectively. Furthermore, we need to consider whether they benefit every worker - not only employees, but also self-employed persons. In other words, we will discuss the personal scope of each provision. Ultimately, we aim to clarify which human rights provisions impose on the states the obligation to ensure collective bargaining for solo self-employed persons.

### A. The European Convention on Human Rights

We start our analysis with a closer look at the European Convention on Human Rights (hereinafter: ECHR), as the most foundational European human rights document. Whereas Art. 11 ECHR explicitly includes the ‘the right to join and form trade unions’, it does not mention the right to bargain collectively. The European Court on Human Rights (hereinafter: ECtHR) had long held that collective bargaining was not indispensable for the effective enjoyment of trade union freedom.^34^ However, in *Demir and Baykara v Turkey* (2008), the Court decided to deviate from its previously established case law.^35^ The case concerned the annulment of a collective agreement concluded for the benefit of municipal civil servants. The collective agreement had already been in place for two years when the Turkish authorities decided that civil servants could not form trade unions according to domestic law. The ECtHR found that the exclusion of civil servants from exercising the right of association according to Art. 11 ECHR was not justified in the case at hand. After considering ‘the perceptive evolution’ in both international and domestic legal systems, it held that the right to collective bargaining constituted an essential element of the freedom of association.^36^ Despite the fact that the decision concerned a collective agreement of employed civil servants, and not self-employed persons, this case is relevant for our analysis as it established a precedent that renders the right to bargain collectively essential to Art. 11 ECHR.

Moving on to the question of the personal scope of Art. 11 ECHR, we note that the wording of the provision benefits ‘everyone’. The ECtHR has also ruled that self-employed persons could, in principle, rely on Art. 11 ECHR.^37^ However, this does not preclude that the state parties may limit the scope of beneficiaries with reference to Art. 11(2) ECHR, which allows the national legislator to restrict the right under certain conditions, namely, when ‘necessary in a democratic society in the interests of national security or public safety, for the prevention of disorder or crime, for the protection of health or morals or for the protection of the rights and freedoms of others.’ The Convention thus allows for a so-called margin of appreciation in the implementation of the rights enshrined in Art. 11 ECHR.

The judgment in *Manole and ‘Romanian Farmers Direct’ v. Romania* (2015) gives some indication as to the ECtHR's interpretation of Art. 11(2) ECHR. The case arose because the Romanian authorities refused to register a union of self-employed farmers. According to Romanian law at the time, only employees and public servants were entitled to set up trade unions.^38^ The Court held that the refusal to register the applicant union had not exceeded the Romanian authorities’ margin of appreciation in the way it secured the right of freedom of association for self-employed farmers, and thus there was no violation of Art. 11 ECHR.^39^ It should be noted, however, that the Court's decision in the *Manole* case seems less extreme considering that the Romanian law under review, whilst prohibiting the formation of trade unions, did allow self-employed farmers to join pre-existing trade unions and to set up trade associations other than trade unions.^40^ In light of this, the Court considered that ‘maintaining the difference between trade unions and other types of associations’ was a legitimate aim according to Art. 11(2) ECHR.^41^ In conclusion, we infer that, even though the Court found no violation in the *Manole* case, a complete denial of collective rights for solo self-employed persons would not be justified.

### B. The European Social Charter

In addition to the ECHR, Austria and Germany have ratified the European Social Charter (hereinafter: ESC),^42^ a human rights document that is equally binding on the state parties, even though the body with powers to interpret it, the European Committee on Social Rights (hereinafter: ESCR), does not issue legally binding decisions. Similar to Art. 11 ECHR, Article 5 ESC prescribes the right for workers and employers to organise. In addition, the ESC includes a separate provision that addresses specifically the right to bargain collectively.^43^ Art. 6(2) ESC states: ‘With a view to ensuring the effective exercise of the right to bargain collectively, the Parties undertake […] to promote, where necessary and appropriate, machinery for voluntary negotiations between employers or employers’ organisations and workers’ organisations, with a view to the regulation of terms and conditions of employment by means of collective agreements […].’

We can therefore directly address the question of whether Art. 6(2) ESC benefits solo self-employed workers. Unlike Art. 11 ECHR, which includes ‘everyone’ in its scope of protection, Art. 6(2) ESC refers to employers and their respective organisations on the one hand, and workers’ organisations on the other. Hence, the question arises as to how the ESCR interprets the wording of the provision, and whether ‘workers’ organisations’ could be interpreted as including associations of solo self-employed workers.

The Committee dealt with this question most extensively in *Irish Congress of Trade Unions v. Ireland* (2018). In this case, a collective agreement that benefitted self-employed voice-over actors was annulled by the Irish competition authority as it was deemed an agreement between ‘undertakings’, and therefore incompatible with the prohibition of price agreements under the Irish Competition Act.^44^ A specificity of the case lies in the fact that the Committee examined two different legal aspects: the legal framework at the time of the complaint and the legal framework at the time of the drafting of the decision by the Committee. The Committee found that the original legal framework was incompatible with Art. 6(2) ESC, since the notion of ‘undertaking’ in the Competition Act was ‘overinclusive’ and amounted to a ban on collective bargaining with respect to remuneration for the self-employed workers at stake.^45^ However, it found that the Competition Act, as amended, did conform with Art. 6(2) ESC.^46^ It explicitly allowed those self-employed working as (voice-over) actors, musicians or freelance journalists to bargain collectively. In addition, it foresaw a mechanism by which self-employed workers in other sectors could be allowed to do so as well. This mechanism was reserved for categories of workers defined under law as either ‘false self-employed workers’ or ‘fully dependent self-employed workers’, and required a decision by the competent Minister to whom a trade union could file an application.^47^

From this decision, we can derive that the Committee does, in principle, allow for a wide interpretation of the term ‘workers’ under Art. 6(2) ESC to include solo self-employed persons.^48^ It has stated that: ‘In establishing the type of collective bargaining that is protected by the Charter, it is not sufficient to rely on distinctions between worker and self-employed, the decisive criterion is rather whether there is an imbalance of power between the providers and engagers of labour.’^49^ At the same time, the Committee has refrained from giving ‘a general definition of how self-employed workers are covered by Art. 6(2) ESC.’^50^ These seemingly contradictory statements can be explained by the existence of Article G of the ESC, which (like Art. 11(2) ECHR) allows state parties to introduce restrictions under certain conditions.^51^ In other words, the question of whether a certain restriction introduced by a state party on the scope of self-employed persons is considered justified, will be evaluated on a case-by-case basis.

### C. The Charter of Fundamental Rights of the EU

Moving from the Council of Europe to the European Union, we will take a look at the Charter of Fundamental Rights of the EU (hereinafter: CFREU).^52^ As a more recently adopted human rights document, the CFREU comprises both the classical liberal rights (Chapter I and II), as well as social rights (Chapter III and IV).^53^ Similar to the ESC, the CFREU contains two provisions that are relevant for collective bargaining: Art. 12 on the freedom of assembly and association is part of Chapter II and prescribes that ‘[e]veryone has the right to freedom of peaceful assembly and to freedom of association at all levels, in particular in political, trade union and civic matters, which implies the right of everyone to form and to join trade unions for the protection of his or her interests.’ In addition, Art. 28 on the right of collective bargaining and action (located in the Chapter IV) provides that ‘[w]orkers and employers, or their respective organisations, have, in accordance with Community law and national laws and practices, the right to negotiate and conclude collective agreements at the appropriate levels and, in cases of conflicts of interest, to take collective action to defend their interests, including strike action.’^54^

Even though Art. 28 CFREU addresses collective bargaining more specifically, a second look at Art. 12 CFREU is warranted due to the diverging formulations of the personal scope of the two provisions. Unlike the ESC, which aligns the personal scope of Art. 5 and Art. 6 ESC, the CFREU grants the right of association to ‘everyone’ (Art. 12 CFREU), whereas the right to collective bargaining and action is reserved for workers and employers and their respective organisations (Art. 28 CFREU). Some scholars argue that, because of the close connection between the two provisions, the rights enshrined in Art. 28 CFREU should also be interpreted as being granted to everyone. Another argument for extending the collective bargaining right under the CFREU can be derived from Art. 52 (3) CFREU. It states that the meaning and the scope of the Charter rights shall be the same as under the relevant provision in the European Convention on Human Rights and, since Art. 11 ECHR implies the right to bargain collectively,^55^ the same material scope shall be attributed to Art. 12 CFREU. ^56^ Finally, we are drawn to take the Explanations attached to the CFREU^57^ into account. They point to the fact that Art. 28 CFREU is based on Art. 6 ESC, and thus provides another line of argumentation in favour of a broad interpretation of the ‘workers’ term under Art. 28 CFREU.^58^

While the Court of Justice of the EU (hereinafter: CJEU) has not yet fully clarified the personal scope of the right of collective bargaining as enshrined in the CFREU, it has developed a line of case law on the autonomous interpretation in EU law of the ‘workers’ definition. In the *Lawrie-Blum v Land Baden Württemberg* decision (1986)*,* it first stated that a ‘worker’ could be any person who performs services (other than merely marginal or ancillary activities) under the direction of another person and receives remuneration in exchange.^59^ The case concerned Ms Lawrie-Blum, a British national, who wanted to avail of the freedom of movement for workers as guaranteed by the Treaties as she sought to be appointed as a trainee teacher in Germany. The Court confirmed her status as a ‘worker’, despite the fact that she was still in training and that teachers were considered civil servants in Germany. The decision established a rather broad interpretation of the term ‘worker’ in the sense that it emphasised the existence of a factual relationship of subordination over more formal characteristics. The term was further developed and applied to different contexts in subsequent decisions.^60^

### D. Interim conclusion

In sum, when comparing the personal scope of the collective bargaining provisions in the ECHR, the ESC and the CFREU, we can conclude that a case-by-case assessment will always be used to determine whether self-employed persons can rely on these human rights provisions. We observe that the wording of the human rights documents examined differs slightly, as do the interpretive approaches adopted by the relevant competent bodies. Whereas the scope of Art. 11 ECHR is broad in its wording, we can only speculate as to whether the ECtHR would find that the Austrian or German legal frameworks are covered by the states’ margins of appreciation. The ECSR is most pronounced in protecting solo self-employed workers, which is noteworthy in light of the fact that the wording of Art. 6(2) ESC raises questions as to the interpretation of the term ‘workers’, as well as on the justification of restrictions according to Article G ESC. For the EU, it remains to be seen whether the Court will also apply the autonomous definition of ‘workers’ developed in the free movement context to Art. 28 CFREU.

Not only does the personal scope of Art. 28 CFREU have be clarified, but also the extent to which the Charter imposes obligations on the Member States. Unlike the ECHR and the ESC, which provide standards against which we can measure the domestic legal frameworks, the CFREU states in Art. 51 that the provisions of the Charter apply to the institutions and bodies of the Union ‘and to the Member States only when they are implementing Union law.’^61^ Therefore, the application of the Charter depends on the extent to which the EU legislator has shaped and will shape labour law in the Member States.^62^ Another caveat stems from the wording of Art. 28 CFREU itself, notably from the fact that the right of collective bargaining is granted only ‘in accordance with Community law and national laws and practices.’^63^ This formulation led to a discussion on the extent to which the EU and national legislators could restrict the rights enshrined in Art. 28.^64^ In the final section, we aim to paint a bigger picture of how the EU shapes collective bargaining in the Member States by looking at potential conflicts between the internal market and the human rights provisions, and at recent advancements on the level of secondary law.

## IV. The European Union: mixed messages on collective bargaining

With the adoption of the Lisbon Treaty the CFREU became part of EU primary law and has fundamentally transformed the EU legal order,^65^ whilst the inclusion of social rights in Chapters III and IV also signifies step in the direction of a more social Europe.^66^ Historically set up as a project to ensure market integration, social goals such as worker protection have only been added to the EU's agenda in a piecemeal fashion. In the early 2000s, the conflict between EU law and the right to collective bargaining became blatantly obvious in a series of rulings (the so-called *Laval* quartet)^67^ in which the CJEU found that the collective actions taken by trade unions were incompatible with the EU's internal market rules, notably the freedom of establishment and the freedom to provide services as guaranteed by Art. 49 and 56 TFEU. These rulings were met with criticism in the literature and, since then, the EU legislator and the Court have shown greater awareness of the autonomy of national collective bargaining systems.^68^ In the following, we will discuss a Treaty provision that proves problematic for collective bargaining by solo-entrepreneurs, in particular.

### A. Collective bargaining as an infringement of EU competition law

With a view to preventing distortions of the internal market, Art. 101 TFEU prohibits, *inter alia*, price agreements between ‘undertakings’ and has been transposed, often word-for-word, into the national law of the Member States.^69^ The question arises as to whether a collective agreement amounts to such a price agreement. In the 1999 *Albany* decision,^70^ the CJEU carved out an exemption from the application of Art. 101 TFEU for collective agreements on behalf of employees. It did so by arguing that the Treaties not only set up a *‘*system ensuring that competition in the internal market is not distorted’ but also a ‘policy in the social sphere.’^71^ But since solo self-employed workers are not formally employed, further clarification was needed on whether they would be qualified as ‘undertakings’ and whether their association would constitute a prohibited price cartel.

The CJEU addressed the issue of collective bargaining by formally self-employed workers in *FNV Kunsten v The Netherlands* (2014).^72^ FNV Kunsten was an employees’ association that concluded a collective agreement which benefitted employed substitute musicians as well as self-employed substitute musicians. The counterpart association terminated the collective agreement, arguing that the collective agreement could not benefit formally self-employed workers who were to be considered ‘undertakings’ in the sense of Article 101 TFEU and therefore precluded from fixing price agreements according to EU competition law. Whilst the Court confirmed, in principle, the characterisation of self-employed service providers as ‘undertakings’,^73^ it went on to argue that this did not apply to ‘false self-employed persons’ in a situation comparable to that of employees.^74^ The Court stated that a service provider cannot be considered an undertaking if he ‘does not determine independently his own conduct on the market, but is entirely dependent on his principal, because he does not bear any of the financial or commercial risks arising out of the latter's activity and operates as an auxiliary within the principal's undertaking.’^75^

Due to its somewhat contracted reasoning, the *FNV Kunsten* decision leaves room for uncertainty. In particular, it leaves us wondering whether the Court has merely provided another specification on the autonomous EU definition of ‘workers’, or whether it has created a new category of ‘false self-employed workers’ in addition to the already existing dichotomy between ‘workers’ and ‘undertakings’.^76^ In 2022, the European Commission provided further clarification by means of the communication of guidelines.^77^ Therein, the Commission defines a solo self-employed person as ‘a person who does not have an employment contract or who is not in an employment relationship, and who relies primarily on his or her own personal labour for the provision of the services concerned’^78^ and specifies the conditions under which their collective agreements are exempted from the application of Union competition law. However, the Guidelines do not provide a binding interpretation of Art. 101 TFEU, but merely a clarification as to how the Commission, as the competent anti-trust authority at EU level, intends to interpret the exemption to Art. 101 TFEU. In particular, they do not bind national competition authorities enforcing competition law against associations of solo self-employed workers at the domestic level.^79^

For now, we can ascertain that EU primary law and its interpretation paint an ambivalent picture, guaranteeing the right of collective bargaining in the CFREU whilst maintaining uncertainty as to the personal scope of the Charter provisions and, at the same time, carving out a (limited) exemption on behalf of solo self-employed persons from the application of competition rules.

### B. Collective bargaining in EU legislation

Having mapped EU primary law, we now move to the level of EU secondary law in order to take two recent advancements of the EU legislator into account: the Adequate Minimum Wage Directive (hereinafter: AMW Directive)^80^ and the Platform Workers Directive.^81^ The former may be seen as part of a paradigm shift towards a more social Europe^82^ and contains provisions to further collective bargaining in general, thus potentially also benefiting solo-entrepreneurs.^83^ The latter has been portrayed as a response to increasing digitalisation of the world of work and may advance the collective bargaining capacity of certain solo self-employed persons, namely, those working in the platform economy.^84^ We discuss these Directives here in the final section of this article because their pending implementation at national level creates a momentum for the national legislature to adapt domestic labour law in a way that takes the European human rights standards, and the interpretative practices of the respective bodies and courts, into account.

The AMW Directive was adopted in 2022 and deploys a two-pronged approach in addressing in-work poverty: It promotes the adoption of statutory minimum wages as well as wage-setting by collective bargaining:^85^ In Art. 5, the AMW Directive requires Member States with statutory minimum wages, such as Germany, to take certain criteria (e.g., the purchasing power and cost of living, the general level of wages, the growth rates of wages or the long-term development of national productivity levels) into account when setting and updating the minimum wage.^86^ Furthermore, they are obliged to use ‘indicative reference values’, such as 60% of the gross median wage, in their assessment of an adequate minimum wage.^87^ In addition, Art. 4 proclaims the general goal of increasing collective bargaining coverage across the Union, e.g., by promoting ‘the building and strengthening of the capacity of the social partners to engage in collective bargaining on wage-setting, in particular at sector or cross-industry level.’^88^ According to Art. 4(2) of the AMW Directive, Member States with a collective bargaining coverage rate of less than 80% – which includes Germany but not Austria^89^ – are obliged to, firstly, provide for a legal framework of enabling conditions for collective bargaining and, secondly, establish an action plan in coordination with the social partners that sets up a timeline as well as measures to increase the collective bargaining coverage rates.

The recitals forming the non-operative part of the AMW Directive speak to the EU legislator’s awareness of the human rights provisions that guarantee the right to collective bargaining: Recital 24, in particular, points, *inter alia*, to Arts. 12 and 28 of the CFREU and the relevant provisions of the ECHR and the ESC.^90^ Nevertheless, the question remains as to whether the AMW Directive promotes collective bargaining only on behalf of employees or also on behalf of solo self-employed persons. In this regard, the scope of the Directive is highly ambiguous in that it applies ‘to workers in the Union who have an employment contract or employment relationship as defined by law, collective agreements or practice in force in each Member State, with consideration to the case-law of the Court of Justice.’ ^91^ It seems that the EU legislator has squared the circle by, on the one hand, safeguarding national autonomy whilst, at the same time, referring to the CJEU case law which has developed an autonomous interpretation of an employment relationship.^92^ A clarification of the personal scope will have to wait as, for now, the CJEU is concerned with the question of whether the adoption of the AMW Directive was within the EU legislator's field of competence at all.^93^ Pending a final decision in that matter, the implementation of the Directive in Austria and Germany is yet unforeseeable.

The Platform Workers Directive was adopted in 2024 and may be conducive to collective bargaining by solo-entrepreneurs who perform work organised through a digital platform.^94^ Its main advancement lies in prescribing the reclassification, under certain conditions, of formally self-employed persons performing platform work as employed workers. It will be up to the digital platforms to prove that their contractual relationship with a self-employed worker is not an employment relationship.^95^ Through the reclassification of their contracts as employment contracts, these workers will thus be included in the collective bargaining systems already set up at national level for employees. Unfortunately, the EU legislator opted for an unclear wording when specifying the type of contractual relationships that are to be reclassified. The wording is reminiscent of that in the AMW Directive in that it combines national qualification criteria with that of the CJEU: According to Art. 5, persons performing platform work ‘shall be legally presumed to be (in) an employment relationship where facts indicating direction and control, in accordance with national law, collective agreements or practice in force in the Member States and with consideration to the case-law of the Court of Justice, are found.’ With a glance at the initial proposal by the Commission, it is evident that the text of the Directive was watered down in the course of the negotiations between the co-legislators. The initial proposal foresaw that the national criteria would be replaced by a harmonised Union-wide test.^96^

A little consolation may be drawn from the fact that the Platform Workers Directive adheres to the right of collective bargaining in two further provisions: In Article 7 (1)(d), it stipulates that digital labour platforms shall be prohibited from using automated data processing in order to predict the exercise of fundamental rights, including the right of collective bargaining. Furthermore, Article 25 requires the Member States to take adequate measures to encourage the exercise of the right to collective bargaining in the platform economy. Thereby, the Directive appeals to the role of the social partners in ensuring the right of collective bargaining through correct determination of the employment status.^97^

## V. Conclusion and recommendations

In this article, we have mapped the legal protection of solo self-employed workers at several levels: national labour law, European human rights standards and EU law. In the first section, we demonstrated that the regulatory frameworks in Austria and Germany need to change in order to allow solo-entrepreneurs to benefit from collective bargaining. We ascertained that these legal changes at domestic level are most evidently required by the right to bargain collectively laid out in Article 6 (2) ESC, which includes solo self-employed persons in the scope of protected workers.

It remains to be seen whether the ECtHR and the CJEU will move in the same direction as the ECSR in the interpretation of the respective provisions in the ECHR and the CFREU. The explanations attached to the CFREU do, in fact, state that the formulation of Art. 28 CFREU was based on Art. 6 ESC.^98^ However, it is unfortunate that the CJEU did not clarify the personal scope of Art. 28 CFREU in its recent *FNV Kunsten* decision, in which it exempted a collective agreement on behalf of solo self-employed persons from the prohibition of price agreements set out in EU competition law. The absence of a discussion of Art. 28 CFREU in that ruling is probably due to the fact that the Court found the Charter inapplicable as it considered the matter as falling outside the scope of EU law.^99^

The AMW Directive and the Platform Workers Directive represent important advancements, not least because the adoption of each legislative act extends the scope of application of the CFREU as more areas of national law are drawn into the scope of the implementation of EU law. However, we can only speculate as to the prospective legal changes that these Directives will trigger at the domestic level. Whereas the AMW Directive is still under review, the time limit for the implementation of the Platform Workers Directive will lapse in 2026. Scholars have noted that the reclassification provision of the Platform Workers Directive gives quite a lot of leeway to the national legislators in defining the criteria that trigger the assumption of an employment relationship.^100^

Therefore, we conclude that Austria and Germany are already, by force of Art. 6 (2) ESC, under the obligation to promote the right to bargain collectively for solo self-employed workers. In this light, we recommend that the Austrian law be amended to include the category of ‘employee-like persons’ in the scope of the Labour Constitution Act by including a legal definition thereof in section 1 of the code. For Germany, we expect that measures will be taken to increase the collective bargaining coverage in general. Additionally, it is essential that the criteria for what makes a worker ‘economically dependent’ according to section 12a TVG will be widened, in order to give a larger proportion of solo self-employed persons the opportunity to bargain collectively.

## Notes

1. See Section 611a (2) of the German Civil Code (short: BGB) and Section 1 of the Austrian Labour Constitution Act (short: ArbVG).

2. C. Kietaibl, *Arbeitsrecht I. Gestalter und Gestaltungsmittel* (new academic press, 12th edn, 2023), 22; M. Gruber-Risak, ‘Persönlich abhängig oder nicht, ist das wirklich noch die Frage? Reflexionen zum Schutzbereich des Arbeitsrechts am Beispiel der Essenszusteller*innen’, (2023) 4 *Juridikum*, 434. For an example of judicial reclassification, see the following judgment of the German Federal Labour Court regarding crowdworkers: Federal Labour Court (BAG), 9 AZR 102/20, 1 December 2020.

3. We rely on the definition introduced by the European Commission: Communication from the Commission Guidelines on the application of Union competition law to collective agreements regarding the working conditions of solo self-employed persons [2022] OJ C 374*,* art 2.

4. Statistik Austria, ‘Arbeitsmarktstatistiken 2024. Ergebnisse der Mikrozensus-Arbeitskräfteerhebung und der Offene-Stellen-Erhebung’ (2025) <www.statistik.at/services/tools/services/publikationen/detail/2168> accessed 14 July 2025, 5.

5. Ibid. 59.

6. The Statistik Austria relies on the Eurostat definition of ‘economically dependent self-employed person’. See: Statistik Austria, ‘Selbstständige Erwerbstätigkeit. Model der Arbeitskräfteerhebung 2017’(2018) <www.statistik.at/fileadmin/publications/Selbstaendige_Erwerbstaetigkeit__Modul_der_AKE_2017.pdf> accessed 14 July 2025, 56.

7. IZA Institute of Labour Economics, ‘Research Report No. 145: Selbstständige Erwerbstätigkeit in Deutschland (Aktualisierung 2024)’, <www.iza.org/publications/r/266/selbststandige-erwerbstatigkeit-in-deutschland-aktualisierung-> accessed 14 July 2025, 18.

8. Ibid. 51.

9. Österreichischer Gewerkschaftsbund, ‘Kollektivvertrag’ (ÖGB website) <www.oegb.at/themen/arbeitsrecht/kollektivvertrag> accessed 14 July 2025.

10. Statistisches Bundesamt, ‘Tarifbindung von Arbeitnehmern’ (DStatis website) <www.destatis.de/DE/Themen/Arbeit/Arbeitsmarkt/Qualitaet-Arbeit/Dimension-5/tarifbindung-arbeitnehmer.html> accessed 17 June 2025. Note, however, that a higher percentage (approx. 65%) can be derived when including companies that align their wage levels with a collective agreement without being bound by it. See: Institut für Arbeitsmarkt und Berufsforschung, ‘Daten zur Tarifbindung und betrieblichen Interessensvertretung’ (IAB website) <www.iab.de/daten/daten-zur-tarifbindung-und-betrieblichen-interessenvertetung> accessed 15 July 2025.

11. The German Statute on Minimum Wages (short: MiLoG) was adopted in 2015. For more background, see: P. Schreiner and S. Sura, ‘Zehn Jahre Mindestlohngesetz’ (2025) NZA, 18–24.

12. See section 1 TVG. See also: Deutscher Gewerkschaftsbund, ‘Was ist ein Tarifvertrag’ (DGB website) <www.dgb.de/service/ratgeber/tarifvertrag> accessed 17 June 2025.

13. Section 5 TVG foresees the possibility for the Ministry of Labour and Social Affairs to declare a collective agreement generally binding, but this occurs rarely in practice. T. Lakies and F. Rödl, ‘§ 5 TVG’ in W. Däubler (eds) *Tarifvertragsgesetz mit Arbeitnehmerentsendegesetz* (Nomos Kommentar 2022), n. 18.

14. See section 2 of the Austrian law on the Chamber of Commerce (short: WKG).

15. Kietaibl (n 2) 207.

16. Ibid. See also section 12 ArbVG.

17. D. Leist and M. Schlachter-Voll, ‘Adequate Remuneration for the self-employed. Legal framework in Germany’, (2025) 1 *Hungarian Labor Law E-Journal* (forthcoming).

18. See section 2 of the Austrian Law on Homeworkers (short: HeimAG).

19. See section 2 of the German Law on Homeworkers (short: HAG).

20. The German Federal Labour Court has included ‘qualified software developer’ in the scope of HAG. See: Federal Labour Court (BAG). 9 AZR 315/15, 14/06/2016.

21. See section 12a TVG.

22. Kietaibl (n 2) 40–42.

23. See section 12a TVG. See also: Leist and Schlachter-Voll (n 17) (forthcoming). G. Spinner, ‘§ 611a BGB’, in M. Henssler and W. Krüger (eds) *Münchener Kommentar zum BGB* (H.C. Beck, 2023).

24. See section 17 HAG.

25. Leist and Schlachter-Voll (n 17) (forthcoming).

26. Ibid.

27. In October 2025, the Austrian Parliament adopted a revision of section 1 of the Labour Constitution Act, to be in force from January 2026. The revision allows for collective bargaining on behalf of a certain group of solo self-employed persons, namely persons with freelance contracts (in German: “freier Dienstvertrag”). Unlike the German TVG, the Austrian Labour Constitution Act does not adress the larger category of employee-like persons. Therefore, solo self-employed persons working on the basis of other types of contracts are still excluded. See section 1 (1) ArbVG, RV 212 BlgNR 28. GP; S. Schwertner, ‘Kollektivverträge für freie Dienstnehmer. Ein Etappensieg?' (2025) 9 *ASoK*, Pages 314–322.

28. See section 43 HeimAG.

29. One of the few homeworkers’ agreements covers those working in the clothing industry. PRO-GE, ‘Heimarbeitsgesamtvertrag für Beschäftigte in der Kleidungsindustrie’ (Pro-Ge website) <www.kollektivvertrag.at/volltext?doc-set-id=SI-2064_de> accessed 21 July 2025.

30. See section 17 of the Journalistic Work act (short: JournG).

31. GPA, ‘Kollektivvertrag für die bei österreichischen Tages- und Wochenzeitungen und deren Nebenausgaben sowie redaktionellen digitalen Angeboten angestellten Redakteure, Redakteursaspiranten und Dienstnehmer des technisch-redaktionellen Dienstes inklusive Gesamtvertrag für Ständig Freie Mitarbeiter’ (GPA website) <www.kollektivvertrag.at/kv/wochenzeitungen-redakteure-u-reporter-ang> accessed 17 June 2025.

32. Leist and Schlachter-Voll (n 17) (forthcoming).

33. A. Seifert, ‘Kollektivverträge für wirtschaftlich abhängige Selbständige und unionsrechtliches Kartellverbot’ (2022) *HSI Working Paper No*, 32; S. Rachor, ‘§ 12a TVG’ in W. Däubler (eds) *Tarifvertragsgesetz mit Arbeitnehmerentsendegesetz* (Nomos Kommentar, 2022), n. 9.

34. *Wilson, National Union of Journalists and Others v the United Kingdom* (App nos 30668/96, 30671/96 and 30678/96) ECHR 2 July 2022 [44]; *Swedish Engine Drivers’ Union v Sweden* (App no 5614/72) ECHR 6 February 1976 [39]; *Gustafsson v Sweden* (App no 15573/89) ECHR 25 April 1996 [45].

35. In *Demir and Baykara,* the ECtHR held that, according to standing case law, only the following three elements were essential to the exercise of the right to association: the right to form and join a trade union, the prohibition of closed-shop agreements and the and the right for a trade union to seek to persuade the employer to hear what it has to say on behalf of its members. See: *Demir and Baykara v Turkey* (App no 34503/97) ECHR 12 November 2008.

36. Ibid. [153].

37. *Vörđur Ólafsson v Iceland* (App no 20161/06) ECHR 27 April 2010 [34][83]. In a case which did not concern self-employed persons, it also noted that Article 11 ‘does not exclude any occupational group from the right of association’. See: *Sindicatul ‘Păstorul Cel Bun’ v Romania* (App no 2330/09) ECHR 9 July 2013 [145].

38. *Manole and ‘Romanian Farmers Direct’ v Romania* (App no 46551/06) ECHR 16 June 2025 [11].

39. Ibid. [70].

40. According to the applicant's submissions, trade associations did not have the same powers as trade unions ‘to negotiate and to submit workers’ demands, inter alia under the right of collective petition.’ Nevertheless, the Court found no reason to doubt that such an association ‘would lack the essential prerogatives for defending its members’ collective interests before the public authorities.’ Ibid. [48][69][72].

41. Ibid. [65].

42. Austria and Germany have both ratified the 1996 revised European Social Charter, but they have not ratified the protocol on the collective complain procedure. See: Council of Europe, ‘Signatures and ratifications’ (Council of Europe website) <www.coe.int/en/web/european-social-charter/signatures-ratifications> accessed 13 April 2025.

43. Austria and Germany have both ratified Art. 5 as well as Art. 6 ESC. See: Council of Europe, ‘Acceptance of provisions of the Revised European Social Charter’ (Council of Europe website) <www.coe.int/en/web/european-social-charter/provisions-of-the-charter> accessed 13 April 2025.

44. The national law on the basis of which the agreement was annulled was adopted in order to implement EU competition law, more precisely Art. 101 TFEU. *Irish Congress of Trade Unions v. Ireland* (Complaint no 123/2016) ESC 12 September 2018 [45][62].

45. Ibid. [98].

46. Ibid. [106][116].

47. In making the decision, the Minister is tasked with ensuring that there is minimal impact on the market and on the State budget, as well as no contravention of any other law, including EU law. See: Ibid. [20][109]

48. Ibid. [35].

49. Ibid. [38].

50. Ibid. [40].

51. Art. G (1) ESC states: ‘The rights and principles set forth in Part I when effectively realised, and their effective exercise as provided for in Part II, shall not be subject to any restrictions or limitations not specified in those parts, except such as are prescribed by law and are necessary in a democratic society for the protection of the rights and freedoms of others or for the protection of public interest, national security, public health, or morals.’

52. Due to the fact that it is not formally legal binding, we exclude the 1989 Community Charter of Fundamental Social Rights for Workers as well as the more recently introduced European Pillar of Social Rights from our analysis. Nevertheless, they may be relevant in the interpretation of the Charter provisions.

53. Whereas the ECHR dates back to 1950 and the ESC to 1961, the CFREU was first adopted in 2000 and became part of EU primary law only with the adoption of the Lisbon Treaty in 2007.

54. Note that, despite the caveats in the wording of the provisions (‘in accordance with EU and national laws’ and ‘at the appropriate levels’), the CJEU recognised Art. 28 as a fundamental right and not as a mere principle in the following decision: Case 271/08 *Germany v Commission (occupational pensions),* ECLI:EU:C:2010:426*.* See: C. Barnard, ‘Article 28’, in S. Peers and others (eds), *The EU Charter of Fundamental Rights. A Commentary* (Hart Publishing, 2014), 780.

55. This has been clarified by the ECtHR decision in *Demir and Baykara v Turkey*, as referenced above.

56. D. Schiek, ‘Towards More Resilience for a Social EU - The Constitutionally Conditioned Internal Market’ (2017) 13 *European Constitutional Law Review*, 611, 633; F. Dorssemont and M. Rocca, ‘Article 28 - Right of Collective Bargaining and Action’, in F. Dorssemont and others (eds), *The Charter of Fundamental Rights of the European Union and the Employment Relation* (Bloomsbury Publishing, 2019), 469.

57. Explanations relating to the Charter of Fundamental Rights [2007] OJ C 303.

58. The ECSR interpreted the personal scope of Art. 6 ESC broadly in the *Irish Congress* case referenced above.

59. Case 66/85, *Lawrie-Blum v Land Baden Württemberg*, ECLI:EU:C:1986:284, para. 19.

60. See, for example, in the context of the EU rules on equal pay for men and women: Case 256/01, *Debra Allonby v Accrington & Rossendale College and Others,* EU:C:2004:18.

61. Art. 51 (1) CFREU.

62. For an elaboration on different interpretations of what falls within the scope of implementation of Union law, see: A. Koukiadaki, ‘Application (Article 51) and Limitations (Article 52(1))’, in F. Dorssemont and others (eds), *The Charter of Fundamental Rights of the European Union and the Employment Relation* (Bloomsbury Publishing, 2019), 140–144; C. Barnard in Peers and others (n 54), 774–777.

63. Art. 28 CREU.

64. Lock states that there would be no point in recognising the right if it were to completely subordinate to EU and national law. According to *Barnard,* this phrase must be read in conjunction with Art. 52 (1) CFREU, which puts limits on the possible restrictions: ‘Subject to the principle of proportionality, limitations may be made only if they are necessary and genuinely meet objectives of general interest recognised by the Union or the need to protect the rights and freedoms of others.’ C. Barnard in Peers and others (n 54), 792; T. Lock, ‘Article 28 CFR’, in M. Kellerbauer and others (eds), *The EU Treaties and the Charter of Fundamental Rights: A Commentary* (OUP, 2019), 2183.

65. G.N. Toggenburg, ‘The EU Charter: Moving from a European Fundamental Rights Ornament to a European Fundamental Rights Order’ in G. Palmisano (eds), *Making the Charter of Fundamental Rights a Living Instrument* (Brill 2014), 4–29.

66. On the discussion on the potential of the Charter to balance the Unions ‘economic constitution’, see for example: D. Schiek (n 56); O. de Schutter, ‘The CFREU and Its Specific Role to Protect Fundamental Social Rights’, in F. Dorssemont and others (eds), *The Charter of Fundamental Rights of the European Union and the Employment Relation* (Bloomsbury Publishing, 2019), 22–32.

67. Case 438/05 *International Transport Workers’ Federation and Finnish Seamen's Union v Viking Line ABP and OÜ Viking Line Eesti,* ECLI:EU:C:2007:772; Case 341/05 *Laval un Partneri Ltd v Svenska Byggnadsarbetareförbundet and Others,* ECLI:EU:C:2007:809; Case 346/06 *Dirk Rüffert v Land Niedersachsen,* ECLI:EU:C:2008:189; Case 319/06 *Commission v Luxembourg*, ECLI:EU:C:2008:350.

68. B. Bercussion, *European Labour Law* (Cambridge Universtity Press, 2009), 705–710; A.C.L. Davies, ‘One Step Forward, Two Steps Back? The Viking and Laval Cases in the ECJ’, (2008) 37 *Industrial Law Journal,* 126–148; H.P. Graver, ‘The Demise of Viking and Laval. The Holship Ruling of the ECtHR and the Protection of fundamental rights in Europe’ (Verfassungsblog website) <www.verfassungsblog.de/holship> accessed 16 June 2025; S. Saurugger and F. Terpan, *The Court of Justice of the European Union and the Politics of Law* (Palgrave, 2017), 187–188.

69. For example, Germany in Section 1 of the Act Against Restraints of Competition (short: GWB) and Austria in Section 1 of its Cartel Act (in German: ‘Kartellgesetz’). Note that the case *Irish Congress* case referenced above also concerned an Irish anti-trust provision that was based on Art. 101 TFEU.

70. Case 67/96 *Albany International BV v Stichting Bedrijfspensioenfonds Textielindustrie*, ECLI:EU:C:1999:430.

71. Ibid. para. 54.

72. Case 413/13 *FNV Kunsten Informatie en Media v Staat der Nederlanden*, ECLI:EU:C:2014:2411.

73. Ibid*.* paras. 26–30.

74. Ibid*.* paras. 31.

75. Ibid*.* para. 33.

76. In favour of the latter interpretation, see: L. Dominik, ‘Ensuring adequate remuneration for vulnerable solo self-employed – EU antitrust prohibition as a limit?’ *Italian Labour Law Journal* (forthcoming); T. Dullinger and M. Risak, ‘The Concept of ‘Worker’ in EU Law. Status Quo and Potential for Change’ (2018) ETUI Research Paper, 23.

77. Communication from the Commission Guidelines on the application of Union competition law to collective agreements regarding the working conditions of solo self-employed persons (2022/C 374/02), 30/09/2022.

78. Ibid. para. 2 (a).

79. Note that Art. 101 TFEU applies only to agreements affecting trade between the Member States. Purely domestic affairs may thus fall outside the Commission's competence. L. Dominik (n 76) (forthcoming).

80. Directive (EU) 2022/2041 of the European Parliament and of the Council of 19 October 2022 on adequate minimum wages in the European Union [2022] OJ L 2022/275.

81. Directive (EU) 2024/2831 of the European Parliament and of the Council of 23 October 2024 on improving working conditions in platform work [2024] OJ L 2024/2831.

82. T. Müller and others, ‘Wages and collective bargaining: the Adequate Minimum Wages Directive as a game changer’, in A. Piasna and S. Theodoropoulou (eds), *Benchmarking Working Europe 2024* (ETUI and ETUC, 2024); J. Cova, ‘The EU directive on fair and adequate minimum wages. Low-pay work, collective bargaining and the prospects for income growth’, (2022) Hertie School Jacques Delors Centre Policy Brief <www.delorscentre.eu/en/publications/fair-and-adequate-minimum-wages> accessed 15 July 2025.

83. For a critical account, see: E. Sjödin, ‘European minimum wage: A Swedish perspective on EU's competence in social policy in the wake of the proposed directive on adequate minimum wages in the EU’, (2022) 13 *European Labour Law Journal,* 273–291.

84. S. Mangold, ‘Platform work and traditional employee protection: The need for alternative legal approaches’, (2024) 15 *European Labour Law Journal*; 726–739, E. Brameshuber and J. Höllwarth, ‘Die EU-Plattformarbeitsrichtlinie. Ein Game-Changer für das Arbeitsrecht?’, (2024) 5 *ASoK*, 170–179.

85. The initial Commission's proposal was structured along a ‘binary divide between countries with statutory minimum wages and countries with collectively negotiated minimum wages.’ See: L. Ratti, ‘The Sword and the Shield: The Directive on Adequate Minimum Wages in the EU’ (2023) 52 *Industrial Law Journal* 477, 483.

86. Art. 5 (1)(2) Directive (EU) 2022/2041.

87. Art. 5 (4) Directive (EU) 2022/2041.

88. Art. 4 (1)(a) Directive (EU) 2022/2041.

89. T. Klein and F. Lentes, ‘EU Mindestlohnrichtlinie (Teil I) Kein Umsetzungsbedarf in Deutschland?’ (2025) 4 *ZESAR*, 116; H. Russegger, ‘Die Mindestlohn-RL der EU: Wie steht es um die Kompetenz der Richtlinie und die Auswirkungen in Österreich?’ (2024) 1 *DRdA*, 16.

90. In addition, it references the ILO Freedom of Association and Protection of the Right to Organise Convention No 87 (1948) and ILO Right to Organise and Collective Bargaining Convention No 98 (1949), the ILO Labour Relations (Public Services) Convention No 151 (1978) and the ILO Collective Bargaining Convention No 154 (1981).

91. Art. 2 Directive (EU) 2022/2041.

92. Recital 21 of Directive (EU) 2022/2041 offers a humble clarification by excluding ‘genuinely self-employed workers’ from the scope of the Directive whilst, at the same time, stating that it will include workers in atypical work arrangements, notably domestic workers, platform workers, voucher-based workers, non-standard workers and the like.

93. Following an action for annulment initiated by the Kingdom of Denmark, Advocate General Emiliou found that the adoption of the AMW Directive was not covered by the EU's competence to regulate working conditions according to 153(1)(b) TFEU because of the explicit exclusion of the matter of pay and the right of association in Art. 153(5) TFEU. See Opinion of AG Emiliou in Case 19/23, *Kingdom of Denmark v European Parliament and Council of the European Union*, ECLI:EU:C:2025:11.

94. It is irrespective whether the work is performed online or in a certain location, as long as it is, at least, mediated by electronic means such as a website or mobile applications. See Art. 2(1) Directive (EU) 2024/2831.

95. Art. 5 Directive (EU) 2024/2831.

96. The initial proposal foresaw five indicative criteria and stipulated that, when two out of the five criteria were fulfilled, the rebuttable presumption would take effect. See Commission (EU), ‘Proposal for a Directive of the European Parliament and of the Council on improving working conditions in platform work’ COM/2021/762 final, Art. 4(2). For commentary see: T. Bourgery-Gonse, ‘At long last, EU countries adopt the platform work directive’ (Euractiv website, 2024) <www.euractiv.com/section/economy-jobs/news/at-long-last-eu-countries-adopt-the-platform-work-directive> accessed 15 July 2025.

97. Art. 25 Directive (EU) 2024/2831.

98. Explanations relating to the Charter of Fundamental Rights [2007] OJ C 303.

99. The Court found that the matter was a ‘purely internal situation’, and that Art. 101 TFEU was not itself applicable. It nevertheless gave guidance on the interpretation of Article 101 TFEU because the Dutch prohibition of cartels was expressly intended to harmonise national competition law with EU law. *FNV Kunsten* (n 72), 17–20.

100. For Germany, see: D. Benkert, ‘Vermutung eines Arbeitsvertrags nach der Plattformarbeits-Richtlinie’ (2024) *NJW Spezial*, 435. F. Lang and H. Reinbach, ‘Umsetzungsfragen zur Plattformarbeitsrichtlinie aus arbeitsrechtlicher Sicht’, (2025) BB, 886. For Austria, see: E. Brameshuber and J. Höllwarth (n 84), 174; F. Pfeiffer, ‘EU-Richtlinie zur Plattformarbeit: Entscheidender Durchbruch oder fauler Kompromiss?’ (AK website, 2024) <www.wien.arbeiterkammer.at/interessenvertretung/eu/infobrief/IB24_3_Artikel02_EU-Richtlinie-zur-Plattformarbeit.html> accessed 15 July 2025.

